# Reduced anti-Müllerian hormone levels in males with inherited bone marrow failure syndromes

**DOI:** 10.1530/EC-23-0510

**Published:** 2024-08-07

**Authors:** Pamela Stratton, Neelam Giri, Sonia Bhala, Martha M Sklavos, Blanche P Alter, Sharon A Savage, Ligia A Pinto

**Affiliations:** 1 Office of the Clinical Director, National Institute of Neurological Disorders and Stroke, National Institutes of Health, Bethesda, Maryland, USA; 2 Clinical Genetics Branch, Division of Cancer Epidemiology and Genetics, National Cancer Institute, National Institutes of Health, Bethesda, Maryland, USA; 3 Leidos Biomedical Research, Frederick National Laboratory for Cancer Research, Frederick, Maryland, USA

**Keywords:** AMH, dyskeratosis congenita, Fanconi anemia, inherited bone marrow failure syndromes, males

## Abstract

Fanconi anemia (FA), dyskeratosis congenita-related telomere biology disorders (DC/TBD), and Diamond–Blackfan anemia (DBA) are inherited bone marrow failure syndromes (IBMFS) with high risks of bone marrow failure, leukemia, and solid tumors. Individuals with FA have reduced fertility. Previously, we showed low levels of anti-Müllerian hormone (AMH), a circulating marker of ovarian reserve, in females with IBMFS. In males, AMH may be a direct marker of Sertoli cell function and an indirect marker of spermatogenesis. In this study, we assessed serum AMH levels in pubertal and postpubertal males with FA, DC/TBD, or DBA and compared this with their unaffected male relatives and unrelated healthy male volunteers. Males with FA had significantly lower levels of AMH (median: 5 ng/mL, range: 1.18–6.75) compared with unaffected male relatives (median: 7.31 ng/mL, range: 3.46–18.82, *P* = 0.03) or healthy male volunteers (median: 7.66 ng/mL, range: 3.3–14.67, *P* = 0.008). Males with DC/TBD had lower levels of AMH (median: 3.76 ng/mL, range: 0–8.9) compared with unaffected relatives (median: 5.31 ng/mL, range: 1.2–17.77, *P* = 0.01) or healthy volunteers (median: 5.995 ng/mL, range: 1.57–14.67, *P* < 0.001). Males with DBA had similar levels of AMH (median: 3.46 ng/mL, range: 2.32–11.85) as unaffected relatives (median: 4.66 ng/mL, range: 0.09–13.51, *P* = 0.56) and healthy volunteers (median: 5.81 ng/mL, range: 1.57–14.67, *P* = 0.10). Our findings suggest a defect in the production of AMH in postpubertal males with FA and DC/TBD, similar to that observed in females. These findings warrant confirmation in larger prospective studies.

## Introduction

Fanconi anemia (FA), dyskeratosis congenita (DC), and Diamond–Blackfan anemia (DBA) are inherited bone marrow failure syndromes (IBMFS) associated with high risks of bone marrow failure (BMF), myelodysplastic syndrome, leukemia, and solid tumors (1, 2, 3). FA is caused by germline pathogenic variants in the FA/BRCA DNA repair pathway associated with BMF at an early age (median age 7 years) and cancer during young adulthood (median age early 30s) (4, 5, 6, 7). Physical anomalies are reported in more than half of individuals with FA. Both males and females with FA have high rates of hypogonadism and infertility (8, 9, 10, 11). DC is the prototype telomere biology disorder (TBD) caused by germline pathogenic variants in telomere biology genes (12). Similar to FA, the risk of BMF and cancer is high in individuals with DC/TBDs (1, 4, 12). DBA, caused by germline variants in genes encoding ribosomal subunits, may present with anemia at birth, with 90% of cases presenting within the first year of life (13, 14). DBA-associated cancers include osteosarcoma and colorectal carcinoma (15). Affected individuals across the IBMFS spectrum are at elevated risk of solid malignancies compared with the general population, although the risk in DBA is not as high as in FA or DC/TBD (4).

Anti-Müllerian hormone (AMH) is a glycoprotein in the transforming growth factor-β (TGF-β) superfamily (16, 17). AMH is a key factor in cell proliferation, cell cycle, cell differentiation, and apoptosis, as well as the apoptotic regression of Müllerian ducts (fallopian tubes, uterus, cervix, and upper 1/3 of the vagina) in male fetuses. The normal male reproductive tract develops *in utero* in response to AMH and testosterone produced by the testes (18). In males, AMH levels drop for a short time after birth, peak within 3–6 months of age, and are maintained consistently throughout infancy to childhood, and then fall prior to puberty (19). AMH levels remain relatively stable in adult males during the reproductive age but decline in elderly men (19, 20, 21).

The concentrations of postneonatal serum AMH and testosterone are inversely related in males (18). High AMH levels in male infants and children can serve as a useful, reliable marker for the presence of testicular tissue when levels of testosterone are low (19, 20). In adult males, AMH has been proposed as a direct marker of Sertoli cell function and an indirect marker of spermatogenesis. Hypogonadism and undescended testes are associated with low AMH levels, suggesting a potential role of AMH in testicular descent; both traits are generally associated with infertility (22, 23, 24, 25). However, a wide overlap of AMH values between controls and infertile men precludes this hormone from being a useful marker of spermatogenesis (26, 27).

The pattern for AMH levels in childhood, adolescence, and adulthood differs between males and females, with serum concentration 5–20 times lower in females than in males (18). In females, AMH is produced exclusively by the granulosa cells of small growing follicles within the ovaries and is correlated with antral follicle count (28).

We have previously shown that AMH levels are significantly reduced in reproductive-age females with FA and DC/TBD (29, 30). The goal of the present study was to assess whether AMH levels are reduced in males with FA, DC/TBD, and DBA.

## Materials and methods

This cross-sectional study includes pubertal and postpubertal males with FA, DC/TBD, and DBA, and their unaffected first-degree male relatives within the well-characterized NCI IBMFS cohort, NIH IRB-approved protocol 02-C-0052 (www.marrowfailure.cancer.gov, Clinicaltrials.gov identifier NCT00027274). All participants and/or their proxies signed written informed consent at enrollment in accordance with the Health and Human Services regulation 45 CFR (Code of Federal Regulations) 46. All clinical and laboratory data were extracted from the study questionnaires and medical records of the participants as previously described (4). Since pubertal and postpubertal healthy male volunteers under age 18 were unavailable, unaffected siblings below age 18 at the time of blood draw served as the controls. Unrelated controls were 25 healthy male volunteers who were participants in the Occupational Health Service Normal Donor Program at the Frederick National Laboratory for Cancer Research (FNLCR) (Frederick, MD, USA). Serum from an additional 11 healthy males was purchased from Equitech-Bio, Inc. The two control groups of unaffected male relatives and healthy unrelated male volunteers were age-matched to patients with FA, DC/TBD, and DBA within 2–3 years when possible. Partnered or married male subjects who reported having children were considered fertile.

### AMH measurement

Serum AMH was measured using the sensitive Gen II AMH ELISA from Beckman Coulter, Inc. at FNLCR according to the manufacturer’s protocol. Excellent intra- and inter-plate reproducibility has been reported for this assay. Reproducibility of the AMH ELISA was confirmed in the FNLCR lab with intra- and inter-plate variability of 10%.

### Statistical analysis

Results are expressed as medians and ranges. Comparison of AMH levels and ages between groups was performed by Mann–Whitney and Kruskal–Wallis tests for non-parametric continuous variables using Stata 16 (StataCorp). *P* < 0.05 was considered significant.

## Results

This study included nine males with FA, 14 unaffected male relatives of patients with FA, 21 males with DC/TBD, 17 unaffected male relatives of patients with DC/TBD, 14 males with DBA, 12 unaffected male relatives of patients with DBA, and 36 unrelated healthy male volunteers (Table 1). There were no statistically significant differences between the median ages of the groups.

**Table 1:** AMH levels in postpubertal males with IBMFS, relatives, and unrelated controls.

	Patients	Relatives	*P*	Healthy volunteers	*P*
**FA cohort characteristics**					
Number of subjects	9	14		19	
Median age, years, when serum drawn (range)	26 (16–37)	31 (14–38)		28 (18–39)	
Median AMH level, ng/mL (range)	5 (1.18–6.75)	7.31 (3.46–18.82)	0.03	7.66 (3.3–14.67)	0.008
**DC/TBD cohort characteristics**					
Number of subjects	21	17		30	
Median age, years, when serum drawn (range)	23 (15–46)	35 (16–48)		28.5 (18–49)	
Median AMH level, ng/mL (range)	3.76 (0–8.9)	5.31 (1.2–17.77)	0.01	5.995 (1.57–14.67)	<0.001
**DBA cohort characteristics**					
Number of subjects	14	12		36	
Median age, years, when serum drawn (range)	31.5 (16–58)	41 (31–62)		39 (18–59)	
Median AMH level, ng/mL (range)	3.46 (2.32–11.85)	4.66 (0.09–13.51)	0.56	5.81 (1.57–14.67)	0.10

**Figure 1:**
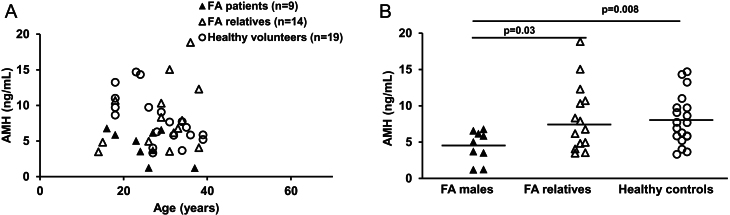
AMH levels in postpubertal male patients with FA (▲) compared with unaffected male FA relatives (Δ) or unrelated male healthy volunteers (⚪). AMH levels for all patients are plotted continuously by age in A and stratified by group in B. Horizontal lines represent median values.

Males with FA had lower AMH levels (median: 5 ng/mL, range: 1.18–6.75 ng/mL) than unaffected relatives (median: 7.31 ng/mL, range: 3.46–18.82 ng/mL; *P* = 0.03) or healthy volunteers (median: 7.66 ng/mL, range: 3.3–14.67 ng/mL; *P* = 0.008) (Fig. 1). Five patients with FA had small/undescended testes, five had undergone hematopoietic cell transplantation (HCT) 7–13 years prior to AMH measurement, and one had head and neck squamous cell cancer (HNSCC) at the time of AMH testing. In total, eight of the nine patients with FA had either hypogonadism, had undergone HCT, or both (Table 2). Several other conditions that might have impacted gonadal function included pituitary stalk interruption (*n* = 1), treated central hypothyroidism (*n* = 2), growth hormone deficiency (*n* = 4), use of androgens for marrow failure (*n* = 1), iron overload (*n* = 1), and use of testosterone (*n* = 1). Only one patient with FA was partnered, and he was infertile on evaluation by fertility specialists. Two other patients with FA have married since the date of AMH testing, but neither have children with one reporting infertility on evaluation by specialists. Overall, none of the patients with FA had fathered a child. In contrast, all eight unaffected relatives who were in relationships or married had children.

**Table 2:** Comorbidities among male inherited bone marrow failure patients and their relatives.

	FA cohort	DC/TBD cohort overall	DC/TBD cohort mild	DC/TBD cohort severe	DBA cohort
Number of patients	9	21	13	8	14
Hematopoietic stem cell transplantation	6	6	3	3	1
Before AMH blood draw	5	0	0	0	0
After AMH blood draw	1	6	3	3	1 (transfusion)
Bone marrow failure	0	5	0	5	4
On steroids	0	0	0	0	6
On androgens	1	4	1	3	0
Chronic transfusion or iron overload	1	5	1	4	2
Cancer	1	2	0	2	0
Before AMH blood draw	1 (head and neck cancer)	2	0	2 (AML, head, and neck cancer)	0
After AMH blood draw	0	0	0	0	1 (lung cancer)
Anomalies	7	2	1	1	0
Small testes	7	1^a^	0	1^a^	0
Undescended testes	0	1^a^	0	1^a^	0
Kartagener’s syndrome	0	1	1	0	0
Pituitary–hypothalamic dysfunction pituitary stalk interruption	1	0	0	0	0
Hypothyroidism	2	1	0	1	0
Growth hormone deficiency	4	0	0	0	1
Low testosterone	0	4	1	3	1
Testosterone treatment	1	0	0	0	1

^a^One subject had small, undescended testes and was hypogonadal.

**Figure 2:**
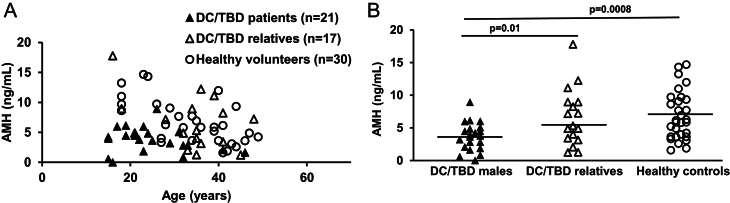
AMH levels in postpubertal male patients with DC/TBD (▲) compared with unaffected male DC/TBD relatives (Δ) or unrelated male healthy volunteers (⚪). AMH levels for all patients are plotted continuously by age in A and stratified by group in B. Horizontal lines represent median values.

Males with DC/TBD had lower AMH levels (median: 3.76 ng/mL, range: 0–8.9 ng/mL) compared with their unaffected relatives (median: 5.31 ng/mL, range: 1.2–17.77 ng/mL; *P* = 0.01) or unrelated healthy volunteers (median: 5.995 ng/mL, range: 1.57–14.67 ng/mL; *P* < 0.001) (Fig. 2). Eight of the 21 patients with DC/TBD had one or more manifestations of severe clinical disease (mucocutaneous triad of nail dystrophy, oral leukoplakia, reticulated skin pigmentation (*n* = 8), severe bone marrow failure (*n* = 7), esophageal stenosis (*n* = 3)). Their AMH levels tended to be lower (median: 2.53 ng/mL, range: 0–6.12 ng/mL) than those with less severe clinical disease (median: 4.21 ng/mL, range: 1.62–8.9 ng/mL, *P* = 0.07). Three patients with DC/TBD with low AMH and severe disease had not attempted fertility. One of these patients, aged 16 years with hypogonadism, had undescended testes and an AMH level of 0 ng/mL; one was in remission 1 year after chemotherapy for acute myeloid leukemia (AMH of 3.2 ng/mL); and one was post-surgery and radiation therapy for HNSCC (AMH of 0.82 ng/mL) (Table 2). None of the patients with DC/TBD had undergone HCT. Unlike patients with FA, all six patients with DC/TBD who were in relationships or married had children; these patients had less severe clinical manifestations of the disease. Since the date of AMH testing, two other patients with less severe disease have married, and both have children. One of the patients had Kartagener’s syndrome and underwent assisted reproductive technology to have children. Other conditions that might impact gonadal function are listed in Table 2. All 13 unaffected relatives who were in long-term relationships or married had children.

**Figure 3:**
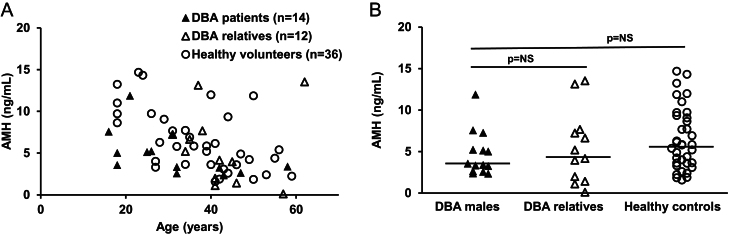
AMH levels in postpubertal male patients with DBA (▲) compared with unaffected male DBA relatives (Δ) and unrelated male healthy volunteers (⚪). AMH levels for all patients are plotted continuously by age in A and stratified by group in B. Horizontal lines represent median values.

AMH levels in males with DBA (median: 3.46 ng/mL, range: 2.3–11.85 ng/mL) were similar to their unaffected relatives (median: 4.66 ng/mL, range: 0.09–13.51 ng/mL; *P* = 0.56) and healthy volunteers (median: 5.81, range: 1.57–14.67; *P* = 0.10) (Fig. 3). Other conditions that might impact gonadal function are listed in Table 2. All eight patients with DBA and all 11 unaffected relatives who were in relationships or married also had children.

AMH levels in all males affected by FA, DC/TBD, and DBA were similar when compared with each other (*P* = 0.5). However, AMH levels in males with FA or DC/TBD were lower in comparison with their unaffected relatives and unrelated controls, while AMH levels in patients with DBA were not significantly different from their relatives and unrelated controls.

## Discussion

We determined that males with FA and DC/TBD had lower AMH levels than age-matched healthy male volunteers and unaffected male relatives. As reported by others, adult males with FA had high rates of hypogonadism, and many had undergone HCT, either of which can be associated with low AMH levels (10, 31, 32). Undergoing HCT prior to puberty appears to have less impact on gonadal function in males or females (32, 33). However, a retrospective series of 41 pediatric males with FA undergoing HCT reported that nearly half became hypogonadal, as shown by declining inhibin B levels after transplantation (34). All males with FA in our study were childless, and most had ‘untested’ fertility. Severe disease in DC/TBD was associated with a trend toward lower AMH levels than DC/TBD with less severe clinical presentations; however, males with severe DC/TBD were younger, unmarried or not partnered, and had ‘untested’ fertility. In contrast, healthier subjects with DC/TBD and those with DBA who were married had children, as did all unaffected relatives who were married. Consistent with prior studies, those with severe clinical manifestations of DC/TBD had autosomal recessive or X-linked recessive inheritance, while most patients with less severe clinical disease had autosomal dominant inheritance (12). We did not have information on fertility testing or offspring for healthy unrelated male controls.

Fertility problems have been reported in both males and females with FA. Males with FA often have primary non-obstructive azoospermia and a Sertoli cell-only phenotype, rendering them infertile in the setting of normal testosterone levels (11, 35). Notably, fertility defects in males with FA appear due to high replicative stress and mutational load both during organogenesis and spermatogenesis (36). In contrast, this study showed that fertility does not appear to be affected in males with DC/TBDs or DBA. However, it is possible that DC/TBDs male fertility may be impacted by the severity and inheritance pattern of the disease, and those with more severe, recessive diseases may have untested fertility due to individuals not living to reproductive age. Cell culture and animal model data suggest that ultra-short telomeres and genes related to the IBMFS syndromes affect gametogenic processes, but the mechanism for this is not well understood in humans (37, 38).

There are limited data on the possible connections between pathogenic variants in IBMFS-related genes and gametogenesis. Male mice with biallelic FA gene mutations have a reduced number of primordial germ cells and decreased proliferation arising from an impaired response to cellular replication stress and increased apoptosis (36, 39). The postnatal testes of male FA mice show dysregulated meiosis, persistent double-strand breaks in meiotic recombination, cells with multipolar spindles, and increased apoptotic bodies. Often a mosaic presentation in the seminiferous tubules of mice results in some tubules containing spermatogonia and others completely devoid, presenting with a Sertoli cell-only phenotype (36). There are no murine studies on fertility in DBA in the current literature, and the biological mechanisms for how ribosomal impairment could affect reproductive function have yet to be elucidated.

There are reports of low AMH levels in men with obesity, diabetes, and metabolic syndrome (40, 41, 42). AMH levels have also been correlated with vitamin D status (43). However, the cross-sectional nature of our study, small sample size, limited laboratory and clinical information on relatives, and the absence of clinical details on healthy controls precluded the evaluation of these factors. Moreover, we did not objectively study fertility or assess the nature of hypogonadism by measuring FSH or inhibin B, or obtaining semen analysis, but noted whether they had children or reported infertility.

The use of AMH levels in males in clinical practice has not been defined and requires further study. Thus far, AMH levels appear to be useful in differentiating non-obstructive azoospermia from obstructive azoospermia, anorchidism from cryptorchidism, and constitutive pubertal delay from congenital hypogonadotropic hypogonadism in prepubertal males (19). Additionally, AMH levels may be useful for assessing testicular damage across the lifespan, pre- and post-chemotherapy interventions, as a potential tumor marker in testicular cancer, and for determining health effects in male relatives of women with polycystic ovarian syndrome (PCOS) (19). Of note, a potential role of AMH in the hypothalamic–pituitary–gonadal axis and neuroendocrine development is suggested by the observation of higher levels in adult male relatives of women with PCOS, although the clinical relevance of this finding is not yet delineated (44, 45).

Our findings suggest a defect in the production of AMH in postpubertal males with FA and DC/TBD, similar to the results in females. This AMH deficiency could be a primary gonadal defect or a consequence of the pathophysiology of the syndromes. Fertility may be impaired in males with FA as it is in females with FA, but fertility is generally not impaired in DC/TBD. Larger longitudinal studies are necessary to confirm these findings and to evaluate the impact of reduced AMH levels in this population.

## Declaration of interest

The authors declare that there is no conflict of interest that could be perceived as prejudicing the impartiality of the study reported. Dr Sklavos completed this work during her post-doctoral fellowship at Dr Pinto’s laboratory at the National Cancer Institute. Dr Sklavos is currently a senior director at Arcellx, Inc.

## Funding

This work was funded by NIH Intramural Research Program, Clinical Center, Clinical Genetics Branch, DCEG, NCI; Program in Reproductive and Adult Endocrinology, NICHD; Leidoshttp://dx.doi.org/10.13039/100015624 Biomedical Research; FNLCR, ClinicalTrials.gov identifier NCT00027274.

## Author contribution statement

PS, NG, MMS, BPA, SAS, and LAP conceived and designed the study. PS, NG, BPA, and SAS collected the clinical data. MMS and LAP performed the laboratory analysis. PS, NG, and SB wrote the paper and provided critical revisions. MMS, BPA, SAS, and LAP reviewed the paper and provided critical revisions.
